# The Potential Roles of Bisphenol A (BPA) Pathogenesis in Autoimmunity

**DOI:** 10.1155/2014/743616

**Published:** 2014-04-07

**Authors:** Datis Kharrazian

**Affiliations:** ^1^1001 Canvasback Court, Carlsbad, CA 92011, USA; ^2^Division of Sciences, Bastyr University California, 4106 Sorrento Valley Boulevard, San Diego, CA 92121, USA

## Abstract

Bisphenol A (BPA) is a monomer found in commonly used consumer plastic goods. Although much attention in recent years has been placed on BPA's impact as an endocrine disruptor, it also appears to activate many immune pathways involved in both autoimmune disease development and autoimmune reactivity provocation. The current scientific literature is void of research papers linking BPA directly to human or animal onset of autoimmunity. This paper explores the impact of BPA on immune reactivity and the potential roles these mechanisms may have on the development or provocation of autoimmune diseases. Potential mechanisms by which BPA may be a contributing risk factor to autoimmune disease development and progression include its impact on hyperprolactinemia, estrogenic immune signaling, cytochrome P450 enzyme disruption, immune signal transduction pathway alteration, cytokine polarization, aryl hydrocarbon activation of Th-17 receptors, molecular mimicry, macrophage activation, lipopolysaccharide activation, and immunoglobulin pathophysiology. In this paper a review of these known autoimmune triggering mechanisms will be correlated with BPA exposure, thereby suggesting that BPA has a role in the pathogenesis of autoimmunity.

## 1. Introduction

Bisphenol A [2, 2 bis(4-hydroxyphenyl) propane; BPA] is a monomer used in the manufacture of polycarbonate plastics. BPA is used in diverse forms of plastic products in the food and electronic industries and in various types of commonly used consumer goods, such as plastic containers, utensils, toys, water bottles, and fax paper. BPA has been shown to leach out of products, and high levels of the monomer have been identified in human and animal samples [1]. The extensive use of BPA-containing products has resulted in high human exposure worldwide [2], with studies reporting that more than 90 percent of the US population has detectable levels in urine samples [3]. It appears that increased temperature leaches BPA into food and water products as does acidic pH of liquids [4]. Additionally, dermal contact with sales receipts and printer paper containing BPA compounds can lead to BPA exposure [5].

BPA has been studied extensively as an endocrine disruptor, and numerous papers have shown how BPA may impact perinatal, childhood, and adult health [6]. BPA has the ability to bind to estrogen receptors and promote both agonist and antagonist activity [7]. It also has the ability to bind to aryl hydrocarbon receptors and exert diverse adverse endocrine effects on human physiology [8]. Its impact on hormone signaling and endocrine dysfunction continues to be an area of research.

BPA also has been shown to have potential adverse neurological effects, especially with respect to fetal brain development and promotion of neurodegenerative diseases [9]. Mice models showing perinatal exposure to BPA inhibits synaptogenesis and affects synaptic structural modification after birth [10]. The impact of BPA on brain health and neurodevelopment also continues to be an area of research.

**Figure 1:**
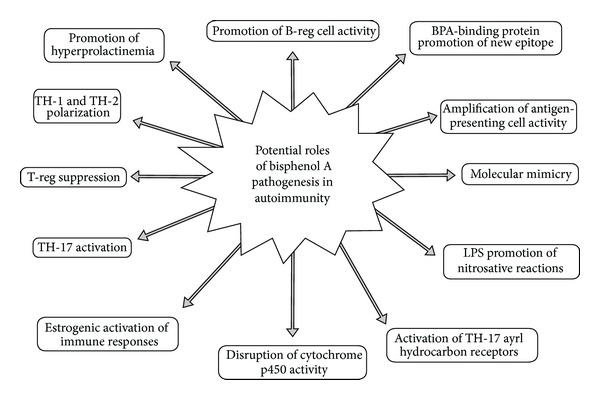
This diagram illustrates the potential mechanisms of bisphenol A's promotion of autoimmunity. BPA: bisphenol A; B-reg cell: regulatory B cell; LPS: lipopolysaccharide; TH: T-helper; T-reg: regulatory T cell.

This paper explores the worldwide exposure to BPA and its potential role in the growing epidemic of autoimmune disease. Although no human or animal studies have been published linking BPA to the onset of autoimmune disease, the potential seems very high due to the physiological influences of BPA and current immunological models regarding loss of self-tolerance and autoimmunity. In addition to known immune mechanisms promoted by BPA that overlap with autoimmune generation, some early evidence also indicates that BPA may contribute to mechanisms that promote autoimmune expression and progression (Figure 1).

## 2. BPA, Hepatic Biotransformation, and Autoimmunity

The hepatic biotransformation of BPA depends on phase I oxidation/reduction involving glutathione and phase II glucuronidation, glutathione, and sulfate conjugation [11]. Healthy humans exposed to BPA appear to have an accumulated body burden of BPA and monitoring studies that measure urinary BPA showed it stored in lipid reservoirs [12]. Despite proper hepatic biotransformation of BPA, the accumulation of BPA in body reservoirs may set the stage for immune reactivity and the onset of autoimmunity. Also, impaired hepatic clearance of circulating immune complexes in response to environmental compounds may induce autoimmunity. In a study of mice exposed to inorganic mercury, those mice that demonstrated reduced hepatic clearance of immune complexes also showed increased levels and altered quality of circulating immune complexes in mercury-induced autoimmunity [13]. Patients with abnormal hepatic biochemistries also have been shown to have a higher frequency of autoimmune disease [14].

A growing body of evidence shows increased toxic loads deplete hepatic tolerance, which leads to over activation of the innate and adaptive immune response and the development of autoimmune disease [15]. Higher BPA concentrations were associated with increased abnormal liver function tests [16]. Animal studies demonstrate that BPA has the ability to generate reactive oxygen species (ROS) and reduce antioxidant reserves and enzymes that are critical for hepatic phase I and II biotransformation, including glutathione, superoxide dismutase, glutathione peroxidase, glutathione S-transferase, glutathione reductase, and catalase activity [17].

**Figure 2:**
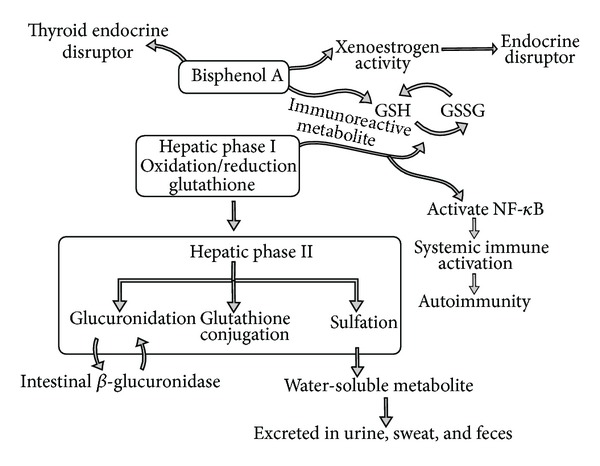
This diagram illustrates the hepatic biotransformation of bisphenol A. GSH: reduced glutathione; GSSG: oxidized glutathione.

BPA disruption of cytochrome P450 enzymes may be a potential mechanism for autoimmune pathophysiology. The cytochrome P450 (CYP) monooxygenases play a crucial role in the liver and various other tissues and are involved with oxidation of organic substances and the bioactivation of drugs and xenoestrogens [18]. CYP activity is necessary for the conversion of xenoestrogens into inactive metabolites that are both noninflammatory and biologically inactive. However, environmental xenoestrogens also have the potential to be metabolized into more reactive and inflammatory metabolites, thereby inducing increased ROS [19]. ROS are involved in apoptosis, activation of antigen presentation cells, and the initiation or amplification of diverse immunologic reactions that may be involved with the pathogenesis of autoimmune disease (Figure 2) [19].

Impairment of hepatic biotransformation of CYP expression may lead to ROS pathophysiology of autoimmunity. ROS have the ability to induce autoreactive molecules that may be involved with both the onset and the exacerbation of autoimmunity [20]. CYP enzymes are involved with metabolizing xenobiotics and producing ROS that may play a role in the pathophysiology of autoimmune disease.

**Figure 3:**
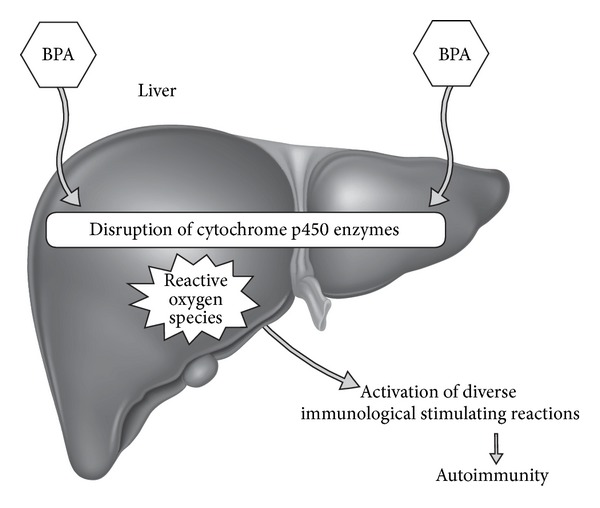
This diagram illustrates how bisphenol A can activate autoimmunity by disrupting cytochrome P450 enzymes. BPA: bisphenol A.

In a study of mice offspring, BPA exposure to 15 and 300 mg/L of drinking water induced cytochrome CYP17 downregulation leading to potential proteomic alterations in immune function [21]. These mechanisms demonstrate the potential for BPA to disrupt proper CYP activity and potentially induce hepatotoxicity by promoting oxidative stress [17]. Increased production of ROS has demonstrated the ability to promote autoimmunity [22]. BPA activity has complex immune-activating reactions throughout the body. The impact of BPA on CYP enzyme expression may be a contributing mechanism to BPA autoimmune pathophysiology (Figure 3).

## 3. BPA Impact on Prolactin Synthesis and Autoimmunity

Although the peptide hormone prolactin is known primarily for its role in lactation, it also plays a critical role in modulating immune and inflammatory responses through various immune signaling pathways [23]. Prolactin has been shown to play significant roles in antigen presenting functions and in the initiation of the response against major histocompatibility complex (MHC) presenting self-antigens as found in autoimmunity [24].

A review of hyperprolactinemia and autoimmunity has found increased prolactin levels associated with production of anti-DNA antibodies, islet cell antibodies, thyroglobulin antibodies, thyroid peroxidase antibodies, adrenocortical antibodies, and transglutaminase antibodies with individuals suffering from systemic lupus erythematosus (SLE), diabetes mellitus type 1, Hashimoto's disease, Addison's disease, and celiac disease [25]. Prolactin has profound immunological stimulating, enhancing, and proliferative responses to antigens and mitogens by promoting increased cytokine activity and immunoglobin production. It also interferes with B cell tolerance and has autoimmune promoting effects [26].

**Figure 4:**
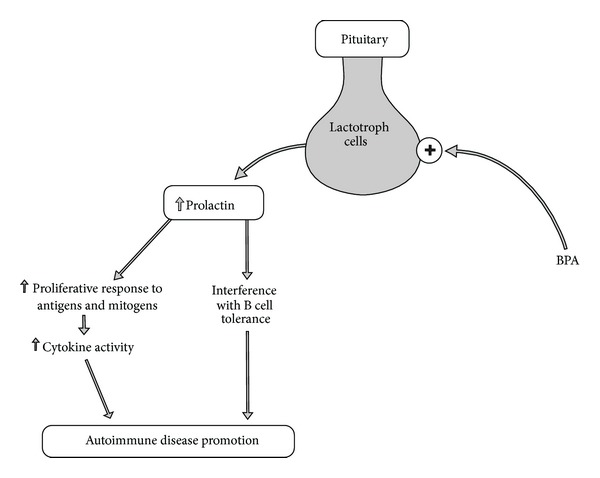
This diagram illustrates how bisphenol A can activate hyperprolactinemia and increase immunostimulatory responses, promoting autoimmunity. BPA: bisphenol A.

BPA is an endocrine disruptor with powerful effects on the pituitary lactotroph cells, which are estrogen responsive and promote prolactin release.* In vitro* and* in vivo* studies have found that BPA mimics estradiol and induces hyperprolactinemia [27]. Therefore, BPA has potential impacts on autoimmune disease activation via its impact on increasing the immunostimulatory response of prolactin (Figure 4).

A link between BPA exposure and increased prolactin levels was found in women workers in occupational settings within one year. They demonstrated marked prolactin level increases and a multivariate analysis found BPA exposure was an independent risk factor for increased serum prolactin levels [28].

The correlation between hyperprolactinemia and autoimmune disease promotion has been reported in the literature in multiple papers during the past 20 years [29]. Additionally, recent evidence has found that BPA has major stimulatory impacts on prolactin release. These correlations strongly suggest that BPA may promote autoimmune pathophysiology by increasing prolactin release that then promotes immune-stimulating activity.

## 4. BPA and Estrogenic Activation of Immune Responses

In addition to the impact of BPA on prolactin release through its estrogenic influence on pituitary cells, BPA also appears to directly affect immune cell signaling pathways and thus immune responses [30]. BPA is classified as an endocrine disruptor in the form of a xenoestrogen and has the potential to mimic estrogen activity throughout the body [31]. This is important because increased circulating estrogens have demonstrated relationships with greater autoimmune activity [32]. Furthermore, epidemiological evidence suggests that the significant increase in the prevalence of autoimmune disease may in part be attributed to environmental estrogens (xenoestrogens). A review of the role of estrogens provides reasonable evidence of an association between xenoestrogen exposure and autoimmune disorders [33].

Various estrogen-promoted mechanisms have been found to trigger autoimmune reactivity. The reticulum transmembrane protein UNC93B1, which is essential for trafficking toll-like receptors (TLRs) from endoplasmic reticulum and is found to play a role in autoimmunity, has been shown to be upregulated by estrogenic signaling [34]. Estrogen activity has also been shown to directly and indirectly stimulate activation-induced deaminase (AID), leading to immune hyperstimulation. AID plays an important role in immune tolerance and the actual elimination of autoantibodies that may impact autoimmune reactivity [35]. Estrogen activity appears to promote signaling of T cell activation in autoimmunity [36]. Estradiol impacts macrophage production of tumor necrosis factor alpha [37]. Treatment of immune cells with estradiol has been shown to increase levels of B cell activating factor (BAFF) mRNA and protein that are associated with increasing severity of autoimmune disease expression [38]. Estrogen activity appears to impact dendritic cell differentiation and interferon production [39]. In summary, estrogen activity appears to have diverse and complex modulatory and stimulating roles in the immune system [40].

**Figure 5:**
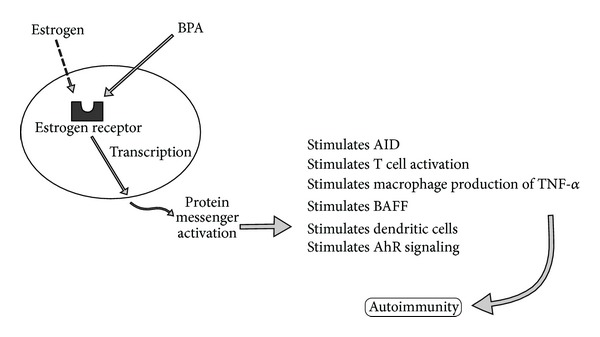
This diagram illustrates how bisphenol A can bind to estrogen receptors and promote estrogenic-mediated autoimmunity. AID: activation-induced deaminase; BAFF: B cell activating factor; BPA: bisphenol A; TNF-alpha: tumor necrosis factor alpha; AhR: aryl hydrocarbon.

Many of these immune-stimulating responses that perpetuate chronic inflammation and autoimmunity may also be potentiated by the estrogenic activity of BPA [41]. BPA stimulates cell proliferation and induced expression of estrogen responsiveness. It also stimulates uterine, vaginal, and mammary growth and differentiation* in vivo* [42]. BPA treatment in mice induced splenocyte proliferation, a shift of cytokine profiles from Th-2 to Th-1 activity, and hyperstimulation of cellular immunity similar to patterns associated with Th-1 dominant autoimmune disease [43]. Overall, BPA has multiple estrogenic mechanisms in promoting abnormal immune responses that include altering T cell subsets, B cell functions, and dendritic cell activity and inducing abnormal immune signaling via its disruptive impact on estrogen receptor signaling, aryl hydrocarbon receptor signaling, and abnormal signaling of peroxisome proliferator-activated nuclear receptors [44]. These BPA estrogenic impacts on virtually all the major cells of the immune system and critical signaling pathways may be one way in which BPA promotes pathogenesis of autoimmunity (Figure 5).

## 5. BPA Impact on Immune Signaling Pathways

BPA has hapten and estrogenic activity, both of which play roles in activating hyperactive immune responses that may occur in autoimmune pathophysiology.

BPA exposure leads to aquatic animal hemocyte immune dysfunction, potentially increasing its role in induced autoimmunity through immune dysregulation. BPA injected into mussels leads to significant lysosomal membrane destabilization and a dramatic decrease in phosphorylation of the stress-activated p38 mitogen-activated protein kinases (MAPKs) and CREB-like transcription factor (cAMP-responsive element-binding protein) in mussels [45]. These results indicate BPA-induced alteration of hemocyte signal transducers and activator of transcription (STAT). These MAPK and STAT pathways are crucial in normal signaling to prevent upregulation of autoreactive T cells found to induce autoimmune inflammatory reactivity [46].

**Figure 6:**
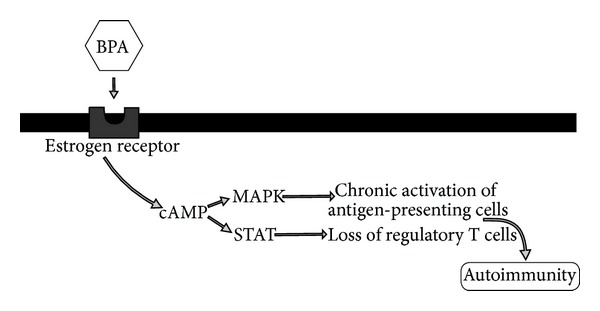
This diagram illustrates how bisphenol A can promote autoimmunity by cellular transcription activation. BPA: bisphenol A; cAMP: adenosine 3′5′-cyclic monophosphate; MARK: mitogen-activated protein kinase; STAT: signal transducer and activator of transcription.

In addition to turning on gene expression of autoreactive T cells, alterations in these MAPK and STAT signaling pathways lead to chronic activation of antigen-presenting cells (APCs), loss of regulatory T cells (CD4+CD25+), apoptosis of APCs, and inhibition of innate and adaptive immunity wind-up found in the pathogenesis of autoimmunity [47]. The signaling pathways that are activated by BPA exposure have been shown to be the exact signaling pathways of molecular processes in autoimmune disease pathophysiology [48]. Therefore, BPA activity as either an estrogenic endocrine disruptor or hapten-activating structure seems to specifically disrupt immune signaling pathways found in autoimmune disease (Figure 6).

## 6. BPA and Cytokine Expression

Cytokines have been shown to play a key role in the pathogenesis of autoimmune disease. The shift of cytokines into Th-1/Th-2 dominance and the IL-17/IL-23 (Th-17) axis has been shown to play pivotal roles in the model of autoimmunity and the breakdown of self-tolerance [49]. BPA has been shown to impact the differentiation processes of the dendritic cells that may cause unintended activation of the immune system in the absence of pathological conditions, thus promoting inappropriate polarization of T cells and cytokine profiles and shifting the immune system into an overzealous immunological state [50]. Additionally, BPA exposure prenatally to mice with oral feeding induced upregulation of Th-1 responses in adulthood [51].

The impact of BPA on naïve immune systems using T cell receptor transgenic mice followed by measurement of cytokine responses to antigens suggest that BPA can augment Th-1 reactions when administered orally in low doses (1.5 mg to 1.8 mg/kg weight) in water. Specifically BPA increased antigen-specific interferon gamma production leading to exaggerated T cell activation and polar Th-1 and Th-2 shifts [52]. These mechanisms associated with interferon have been shown to play powerful effector roles in the pathogenesis of autoimmunity, especially system autoimmunity such as systemic lupus erythematosus [53].

Animal studies have also shown that BPA exposure promotes cytokine inflammatory shifts associated with potential autoimmune development. BPA administered to mice in drinking water produced significant shifts of lymphocytes subpopulations. The production of inflammatory Th-1 type cytokines (IFN-gamma) was induced while Th-2 cytokine (IL-4) was suppressed with BPA treatment, promoting the transcription of IRF-1. The mRNA expression of GATA-3 was inhibited in BPA-treated groups in dosages of 0.015, 1.5, and 30 mg/mL for 4 weeks [54]. These responses indicated that BPA has the potential to induce Th-1 polar shifts of transcription factor that lead to exaggerated cellular immune responses leading to an exaggerated Th-1 immune response. The suppression of GATA-3 transcription factors and T cell polarization favoring a Th-1 bias has been shown to be an immune mechanism of multiple sclerosis autoimmunity in animals [55].

A study comparing the effect of BPA exposure on cytokine activity in adulthood and prenatally demonstrated that in adulthood exposure to BPA significantly promoted antigen-stimulated production of IL-4, IL-10, and IL-13, but not IFN-gamma. However, mice exposed prenatally to BPA showed increased production of not only IL-4 but also IFN-gamma. The percentages of T regulatory function (CD4+CD25+) were decreased in both groups exposed to BPA [56]. Loss of regulatory T cell function promotes abnormal cytokine shifts that occur in autoimmune diseases [57]. Suppression of regulatory T cell function leading to impaired cytokine modulation may be part of the immunopathology of BPA autoimmune development.

**Figure 7:**
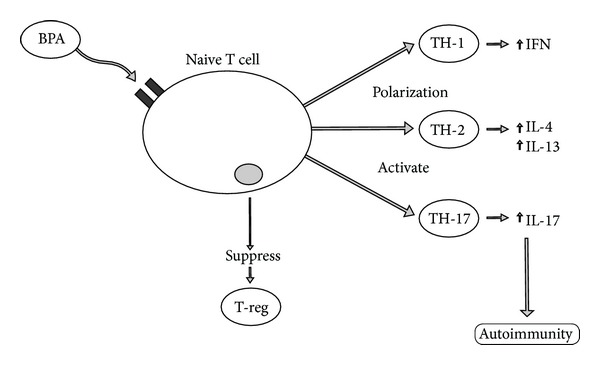
This diagram illustrates how bisphenol A can induce T cell shifts, promoting autoimmunity. BPA: bisphenol A; IFN: interferon; IL: interleukin; TH: T-helper; T-reg: regulatory T cell.

The delicate interplay between Th-1, Th-2, and Th-17 expression appear to be a key factor in autoimmune pathophysiology. Evidence indicates that BPA may induce polarity in this delicate balance and trigger inflammatory reactions, potentially leading to loss of self-tolerance as noted in subsequent paragraphs. The impact of BPA on the pathogenesis of abnormal cytokine shifts most likely occurs from complex web-like reactions. BPA's role as both a hapten and estrogenic endocrine disruptor appears to promote multiple interwoven pathways involved in adverse cytokine shifts that may play a role in autoimmune pathogenesis (Figure 7).

## 7. BPA and Lipopolysaccharide-Induced Nitric Oxide Production

Bacterial translocation of lipopolysaccharides (LPS) has the ability to activate oxidative and nitrosamine stress pathways associated with the inflammatory responses and pathophysiology of autoimmune responses [58]. BPA directly impacts LPS activation of these pathways, and the role of BPA on LPS activation could likewise play a role in abnormal immune reactivity [59].

**Figure 8:**
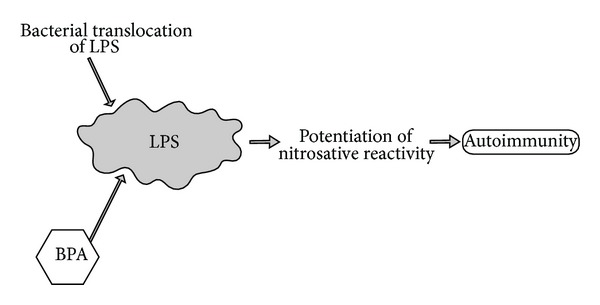
This diagram illustrates how bisphenol A can promote lipopolysaccharide inflammatory sequelae. BPA: bisphenol A; LPS: lipopolysaccharide.

Additionally, decreased activation of LPS-induced inflammatory reactions has also demonstrated a reduction in inflammatory sequelae of autoimmune cytokine and chemokine expression. Specifically, mice injected with BPA exhibited increased endotoxin-induced macrophage activation, suggesting that BPA may potentiate infectious autoimmune inflammatory reactions via enhanced tumor necrosis factor and nitric oxide reactivity [60]. Therefore, LPS-induced expression of nitrosative stress reactivity may be a key factor in BPA-promoted models of autoimmunity associated with infectious autoimmune reactions (Figure 8).

## 8. BPA Impacts on Antigen-Presenting Cell Reactivity

Antigen-presenting cells such as dendritic cells and macrophages appear to play a potential role with BPA and autoimmune reactivity. Dendritic cells (DCs) are important antigen-presenting cells that play a critical role in adaptive immunity due to their ability to activate naïve T cells, which, when overzealous, could promote autoimmune activity [61]. DCs promote the expressions of Th-1, Th-2, or Th-17 cells that can be switched to express autoimmune inflammatory cascades [62]. DCs exposed to BPA in combination with tumor necrosis factor alpha promote CC chemokine ligand 1 (CCL1) signaling, a chemokine that is known to trigger chemotaxis of CCr8 expressing Th-2 and a subset of T regulatory cells, thereby promoting higher levels of IL-10 relative to those of IL-12p70 on CD40 ligation and preferentially inducing Th-2 deviation [63]. These variant responses from DCs exposed to BPA may play a role in autoimmunity.

Macrophage modulation of nitric oxide release is also critical for the regulation of apoptosis and differentiation of T cells that may lead to progression of autoimmune disease [64]. Additionally, BPA exposure has the ability to exert disruptive effects on macrophages by binding to estrogen receptors and leading to alteration of nitric oxide production and TNF-alpha synthesis in the homeostasis of TH-1 and TH-2 activity [65]. These macrophage expressions from BPA may promote immunological shifts that occur with autoimmunity, linking BPA's potential role to abnormal antigen-presenting cell responses.

## 9. BPA Effects on Immunoglobulin Activity

Increased immunoglobulin reactivity from endocrine disruptors such as BPA may raise concerns about immune hyperactivity associated with autoimmune immunopathology. The activation of immunoglobulins has a potential to promote inflammatory or anti-inflammatory activities through the activation of regulatory B (Breg) cells. Recent research in mice has shown that when B cell expression shifts into IL-10 production, there are suppressive effects on inflammatory responses. However, promotion of IgE-producing B cells plays a direct role in promoting inflammatory responses and the development of immune upregulation associated with most underlying inflammatory conditions, such as allergies and autoimmunity [66].

**Figure 9:**
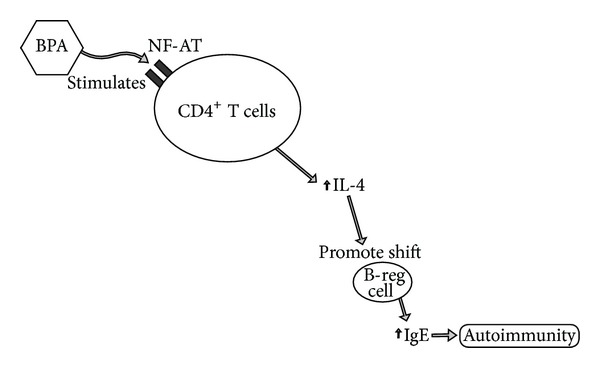
This diagram illustrates how bisphenol A can impact immunoglobulin-promoted autoimmunity. BPA: bisphenol A; B-reg cell: regulatory B cell; IL: interleukin; IgE: immunoglobulin E; NF-AT: Ca2^+^/calcineurin-dependent nuclear factor binding sites.

Recent research has shown that BPA has a direct impact on increasing immunoglobulin expression into the inflammatory IgE response, thereby potentially promoting an inflammatory cascade in autoimmunity. Specifically, exposure to BPA was shown to increase IL-4 production in CD4^+^ T cells and antigen-specific IgE levels in sera via the stimulation of Ca^2+^/calcineurin-dependent nuclear factor of activated T cells binding sites (NF-AT) [67]. These immune responses have the ability to potentiate allergies and autoimmune reactions in those with autoimmunity. Increased levels of IgE may play a direct role in promoting the inflammatory responses found in autoimmunity [68]. The potential for BPA to increase IL-4 and promote a shift of Breg cells into IgE production may be a mechanism for BPA autoimmune promotion (Figure 9).

In a murine model for SLE, animals implanted with BPA specifically demonstrated B cell activation and promotion of autoimmune disease such as lupus nephritis. BPA implantation enhanced autoantibody production by B1 cells both* in vitro* and* in vivo* in murine models of SLE. The study researchers suggested that BPA exacerbates preexisting autoimmune diseases such as SLE and that continued exposure to endocrine disruptors may potentiate the incidence and severity of autoimmune diseases [69].

Evidence of BPA on expressing B cell activity towards inflammatory expression and autoimmune development may partly explain the complex immune web reactions of this endocrine disruptor. Although inflammatory immunoglobulin reactivity may have a role to play in autoimmune expression, it is most likely part of a larger complex immune reaction that is linked to this very reactive endocrine disruptor.

## 10. BPA-Binding Protein: A Potential New Epitope

**Figure 10:**
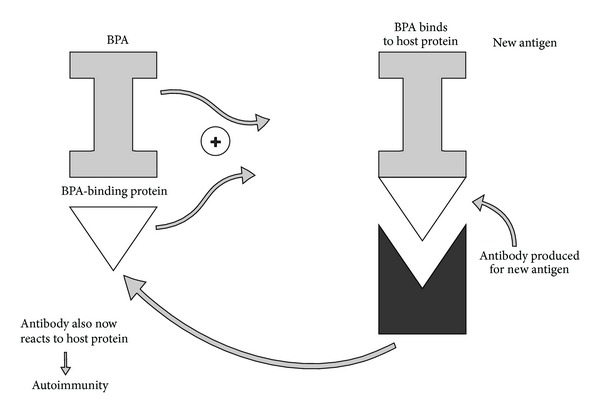
This diagram illustrates how bisphenol A can bind to the host protein, leading to a new epitope reaction against the host protein, resulting in autoimmunity. BPA: bisphenol A.

BPA binds to host protein, potentially creating a new epitope for immune reactivity. BPA binds to protein disulfide isomerase (PDI), also known as BPA-binding protein [70], a multifunctional protein involved in diverse cellular functions. This binding protein has been associated with endocrine disruptor mechanisms involving BPA [71]. The binding of environmental BPA to host protein may lead to self-tissue, antigen-antibody interactions associated with environmentally induced molecular mimicry. Autoimmune molecular mimicry requires the similarities of surface topologies leading to antigenic combining sites [72]. The binding of BPA to PDI in host has the potential to lead to new protein epitope activation of autoimmunity (Figure 10).

## 11. BPA and Autoimmune Molecular Mimicry

**Figure 11:**
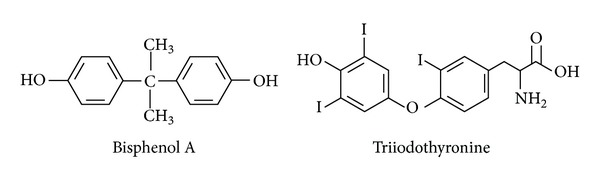
This diagram illustrates the structural similarity between bisphenol A and triiodothyronine, leading to potential cross-reactivity.

BPA and triiodothyronine (T3) possess such a degree of molecular structure similarity that BPA may act as an antagonist compound on T3 receptor sites [73]. When compounds have structural similarity, it may potentially lead to autoimmune cross-reactivity with antigen-antibody complexes [74]. In particular, environmental compounds such as hydrocarbon rings found both on BPA and T3 with anchor ring like similarities may induce mimicry [75]. A potential mechanism for the role of BPA in autoimmunity may be structural molecular mimicry, in particular with thyroid hormones (Figure 11).

## 12. BPA and TH-17 Aryl Hydrocarbon Receptors

**Figure 12:**
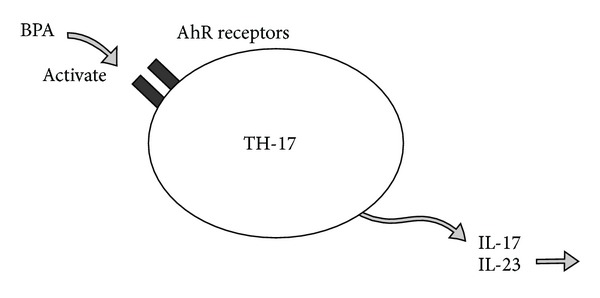
This diagram illustrates how bisphenol A can activate autoimmunity by inducing mRNA expression on aryl hydrocarbon receptors on TH-17 cells. AhR: aryl hydrocarbon; BPA: bisphenol A; IL: interleukin; TH: T-helper.

Aryl hydrocarbon receptors (AhR) are involved with regulating immune responses and the development of TH-17 cells, which are key effector T cells in a variety of human autoimmune diseases. [76] Exposure to low dose BPA has been shown to upregulate mRNA expressions of AhR. AhR activation of TH-17 by BPA may potentiate autoimmunity. The role of chemical contamination and its ability to prompt AhR receptor activation of TH-17 have already been investigated in allergic and autoimmune diseases [77]. Although direct evidence has not been investigated for the role of BPA on AhR activation of TH-17 autoimmune reactivity, the potential mechanism may exist (Figure 12).

## 13. Conclusion

**Figure 13:**
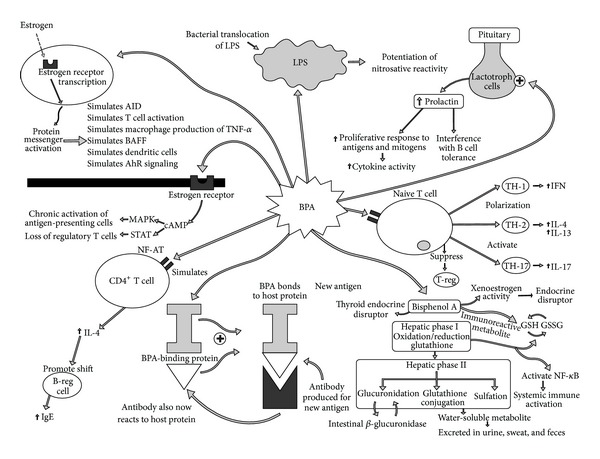
Potential of various autoimmune mechanisms from bisphenol A.

With the growing epidemic of autoimmune disease worldwide and the extensive use of consumer goods containing BPA, we must examine the risk of BPA as a potential triggering compound in autoimmune disease. Although no specific evidence has linked human or animal autoimmune disease development to BPA exposure, many of the mechanisms known to exist in autoimmune pathophysiology also appear to exist with immune reactivity from BPA exposure (Figure 13). Further investigation needs to be conducted correlating autoimmune disease development to BPA exposure. Additionally, the impact of BPA exposure on those already suffering from autoimmunity needs to be investigated further based on potential overlapping pathophysiology.
